# Host-Seeking Activity of Bluetongue Virus Vectors: Endo/Exophagy and Circadian Rhythm of *Culicoides* in Western Europe

**DOI:** 10.1371/journal.pone.0048120

**Published:** 2012-10-29

**Authors:** Elvina Viennet, Claire Garros, Ignace Rakotoarivony, Xavier Allène, Laëtitia Gardès, Jonathan Lhoir, Ivanna Fuentes, Roger Venail, Didier Crochet, Renaud Lancelot, Mickael Riou, Catherine Moulia, Thierry Baldet, Thomas Balenghien

**Affiliations:** 1 CIRAD, UMR Contrôle des Maladies, Montpellier, France; 2 EID-Méditerranée, Montpellier, France; 3 INRA, UE1277 PFIE, Plate Forme d’Infectiologie Expérimentale, Nouzilly, France; 4 Université de Montpellier 2, ISEM équipe « Génomique interactive », Montpellier, France; University of Liverpool, United Kingdom

## Abstract

Feeding success of free-living hematophagous insects depends on their ability to be active when hosts are available and to reach places where hosts are accessible. When the hematophagous insect is a vector of pathogens, determining the components of host-seeking behavior is of primary interest for the assessment of transmission risk. Our aim was to describe endo/exophagy and circadian host-seeking activity of Palaearctic *Culicoides* species, which are major biting pests and arbovirus vectors, using drop traps and suction traps baited with four sheep, as bluetongue virus hosts. Collections were carried out in the field, a largely-open stable and an enclosed stable during six collection periods of 24 hours in April/May, in late June and in September/October 2010 in western France. A total of 986 *Culicoides* belonging to 13 species, mainly *C. brunnicans* and *C. obsoletus*, was collected on animal baits. *Culicoides brunnicans* was clearly exophagic, whereas *C. obsoletus* was able to enter stables. *Culicoides brunnicans* exhibited a bimodal pattern of host-seeking activity with peaks just after sunrise and sunset. *Culicoides obsoletus* was active before sunset in spring and autumn and after sunset in summer, thus illustrating influence of other parameters than light, especially temperature. Description of host-seeking behaviors allowed us to discuss control strategies for transmission of *Culicoides*-borne pathogens, such as bluetongue virus. However, practical vector-control recommendations are difficult to provide because of the variation in the degree of endophagy and time of host-seeking activity.

## Introduction

Host-parasite systems are subject to opposing selective processes: on the one hand, parasite fitness is increased by a higher frequency of encounters with hosts and on the other hand, host fitness is increased by avoiding these contacts [1]. Feeding success of free-living hematophagous insects, which can be considered parasites of vertebrates with periodic and brief contacts, depends on their ability to be active when hosts are available and to reach places where hosts are accessible [2]. Circadian and seasonal activities of insect are regulated by endogenous oscillators which are initiated by natural diel alternation of light and darkness [3]. These biological clocks enable organisms to anticipate variations of biotic and abiotic factors associated with seasonal or diel progression rather than follow them [3], [4]. Diel timing of host-seeking activity should be selected to ensure temporal encounter of the insect and host and to minimize the risk of dying during host-seeking activities. Similarly, endophagy, defined as the trend of obtaining a blood meal within a man-made structure [3], can be regarded as an adaptation of hematophagous insect to reach their hosts.

The description of circadian activity and endo/exophagy of hematophagous insects could have practical implications for the control of diseases induced by transmitted pathogens. A well-known example is stabling horses at night in South Africa where *Culicoides imicola* Kieffer is known to be nocturnal and mainly exophagic [5], [6]. This vector-control strategy has proven to be an environmentally-friendly and inexpensive method to prevent African horse sickness (AHS) by reducing bites of the main vector. From 2006 to 2008, Europe faced a huge epizootic of bluetongue virus serotype 8 (BTV8) transmission, leading to disastrous sanitary consequences in domestic ruminant populations and to important disruptions in animal trade [7]. European regulations recommend the stabling of animals during *Culicoides* activity to reduce BTV transmission (EU Council Directive 2000/75/EC). The direct costs due to indoor housing of animals in The Netherlands in 2006, which was compulsory in restriction zones, were estimated to 18 million Euros, *i.e.* 55% of total economic impact [8]. However, endo/exophagy of Palaearctic *Culicoides* was not precisely described and then benefits of this housing strategy were uncertain. The recent emergence of a novel Orthobunyavirus, named Schmallenberg virus [9], in European ruminant populations highlighted the crucial need of address this issue.

Endophagous behavior of Palaearctic species was reported first by Overgaard Nielsen and Christensen [10] in Denmark. In late 2006, comparing UV-light trap collections inside and outside sheds in The Netherlands, Meiswinkel *et al.* [11] collected threefold more *Culicoides* outside than inside, where cattle were kept for the night. The species collected mainly inside, *i.e. Culicoides obsoletus* Meigen*/Culicoides scoticus* Downes and Kettle, *Culicoides dewulfi* Goetghebuer and *Culicoides chiopterus* (Meigen), were suspected to be involved in BTV8 transmission [11]. Using the same method in France at the same period, Baldet *et al.* [12] collected mainly the same species inside stables, but in greater number inside than outside. Authors conceded that the results were difficult to compare and interpret because of the variation in building openings and in cattle abundance close to the trap in the different collection sites [12]. With a standardized approach, Baylis et al. [13] highlighted that the presence of animals and the opening of stable increased the indoor number of *Culicoides* collected by UV-light trap. Moreover, authors showed that the decrease of *Culicoides* number between outside and inside traps is greater in summer (6.5 fold) than in autumn (3 fold) [13]. Unfortunately, all these studies used UV-light traps to assess *Culicoides* abundance, which do not assess correctly the biting rate on animals [14]–[16]. Indeed, light attraction might lead to an overestimation of the *Culicoides* endophagy, due to an higher trapping efficiency when light traps are set inside stables, rather than outside [17].

Similarly, circadian cycles of Palaearctic species are poorly described. It is widely assumed that *Culicoides* are mostly crepuscular and may continue to be active throughout the night. However, many species are also troublesome in the day displaying two biting peaks: one after sunrise and the other close to sunset. Hours of midge attacks can lengthen when low-light overcast conditions prevail, leading to biting throughout the day in both open (*Culicoides impunctatus* Goetghebuer) and forested environments (*C. obsoletus*) [17]. However, only a few studies have described rigorously *Culicoides* nycthemeral cycles with consecutive 24 h collections [18], [19], and only one has utilized host-seeking behavior of European *Culicoides* [20]. Using horse-baited collections, the latter found that the largest number of *C. obsoletus* was collected at sunset, far less at sunrise and occasionally in the afternoon and night.

The aim of this study was to describe circadian host-seeking activity and endo/exophagous behavior of Palaearctic *Culicoides* using host-baited trap collections. As insect behavior is directly affected by geophysical (sun and moon light cycles) and climatic (temperature, humidity, wind) factors, we carried out collections in three different periods of the year.

## Results

### Climatic Data

**Figure 1:**
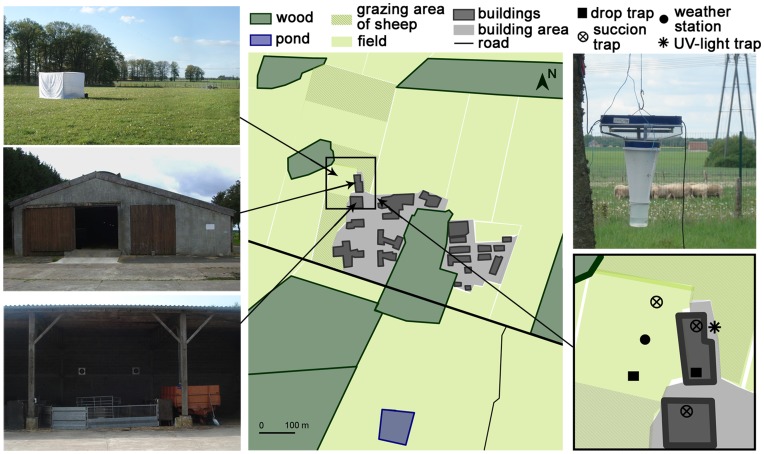
Climatic conditions in the study site. A. Ombrothermic diagram for comparison between 2010 and the 1971–2000 period (data from Météo-France station of Parcay-Meslay); B. Boxplots of temperature (°C) and relative humidity (%) recorded by data loggers at each location during the 15 collection sessions; C. Boxplots of mean and maximum wind speed (km/h) recorded by the local weather station during each of the 6 summer collections.

Climate at the study site was oceanic, with a mean annual temperature of 11.4°C, thermal amplitude of 14.9°C and annual rainfall of 694 mm (Météo-France data, 1971–2000). In 2010, annual rainfall was lower than for the 1971–2000 period (586 mm), with deficit in rainfall during the first part of the year (179 mm between January and May 2010 *versus* 298 mm for the reference period). Temperatures were generally close to normal values in 2010, with colder values in January and December and warmer in June and July (Fig. 1A).

During collection sessions, temperatures were higher in summer than in autumn and spring, with smaller amplitude in autumn, whereas humidity was higher in autumn than in summer and spring (Fig. 1B). The temperature decreased gradually from indoors to outdoors, while humidity showed the inverse tendency. In autumn, however, the relative humidity was the highest in the largely-open stable (Fig. 1B). Amplitudes of temperature and humidity were higher outdoors than in other locations, with outlier observations, especially in autumn, probably due to direct exposure of recording devices to the sun. Wind speeds were correlated between locations, with an expected wind gradient from outdoors to indoors, with an intermediate situation for the largely-open stable (Fig. 1C).

### Diversity and Seasonality

During the 18 collection sessions, a total of 986 *Culicoides* (948 females and 38 males) belonging to 13 species was collected in host-baited traps (drop trap and suction trap) and 539 *Culicoides* (451 females and 88 males) belonging to 19 species in the UV-light/suction trap (Table 1). Molecular assays were performed on 407 *Culicoides* females morphologically identified as belonging to the Obsoletus Complex. The sample contained 349 *C. obsoletus*, 25 *C. scoticus*, 6 *C. dewulfi* and 1 *C. chiopterus*; 26 individuals were not identified. Only 4% of *Culicoides* collected in host-baited traps were males – mainly the dominant species *Culicoides brunnicans* Edwards collected by the drop trap – whereas 20% of *Culicoides* were males in UV-light/suction trap. A total of 84 gravid females was collected: all in the suction trap mainly in summer (47%) and most were *C. obsoletus* (76%). Blood-fed females were collected by all traps, but mainly by the drop trap (197 *versus* 4 in suction trap and 9 in the UV-light/suction trap). The majority of blood-fed females (196/210) belonged to the dominant species *C. brunnicans* and *C. obsoletus*; the engorgement rate in the drop trap was 28% for *C. brunnicans* and 65% for *C. obsoletus*.

**Table 1:** Numbers of *Culicoides* collected over 18 sessions in spring, summer and autumn by host-baited traps and UV-light/suction trap.

Location		R^1^	Species	Host-baited traps																		UV-light/suction trap										
				Total		Suction Trap								Drop Trap								Total		Spring			Summer			Autumn		
						Spring		Summer			Autumn			Spring			Summer			Autumn												
				**F** 2	**M**	**F**	***P***	**F**	***P***	**M**	**F**	***P***	**M**	**F**	***P***	**M**	**F**	***P***	**M**	**F**	***P***	**F**	**M**	**F**	***P***	**M**	**F**	***P***	**M**	**F**	***P***	**M**
OUTDOORS	Field	*1*	*C. brunnicans*	592	24	111	*11*							475	*50*	24	6	*67*				140	62	126	*5*	62	14	*93*				
		*2*	*C. obsoletus*	193	3	31	*55*	61	*54*		27	*63*	2	17	*65*		42	*60*	1	15	*40*	186	16	17	*59*		95	*43*	3	74	*38*	13
		*3*	*C. scoticus*	43		29	*81*	5	*40*		2			6	*83*					1		29	2	18	*33*		8	*75*		3		2
		*4*	*C. punctatus*	4	1	1		1					1				1			1		27	4				7	*57*		20	*80*	4
		*5*	*C. festivipennis*																			22					11	*100*		11	*91*	
		*6*	*C. pulicaris*	8		2		1						5	*40*							12	1				12	*50*	1			
		*7*	*C. dewulfi*	16		5		3			6	*33*		1			1															
		*8*	*C. chiopterus*	10		4		5	*100*								1															
		*9*	*C. poperinghensis*	2													2					6		6								
		*10*	*C. achrayi*																			4					4					
		*10*	*C. lupicaris*	1				1														3		2			1					
		*10*	*C. vexans*	3		2								1								1		1								
		*13*	*C. nubeculosus*	3							1						1			1												
		*13*	*C. simulator*																			3					3					
		*15*	*C. kibunensis*																			2					2					
		*15*	*C. santonicus*	1		1																1	2	1		2						
		*17*	*C. fascipennis*																			1								1		
		*17*	*C. pictipennis*																			1		1								
		*17*	*C. salinarius*																			1								1		
		*17*	*C. puncticollis*																				1									1
			Obsoletus Complex	24		3		9	*67*		2			9	*55*		1					10		1			6	*83*		3		
			Pulicaris Group		1								1																			
	**Total Field**			**900**	**29**	**189**		**86**			**38**		**4**	**514**		**24**	**55**		**1**	**18**		**451**	**88**	**174**		**64**	**164**		**4**	**113**		**20**
INDOORS	Largely open stable	*1*	*C. brunnicans*	1		1																										
		*2*	*C. obsoletus*	13	2	1		3			9	*56*	2																			
		*3*	*C. scoticus*	4		2					2																					
		*7*	*C. dewulfi*	1							1																					
		*17*	*C. newsteadi*	1				1																								
			Obsoletus Complex	1				1																								
	**Total Largely open stable**			**21**	**2**	**4**		**5**			**12**		**2**	***–***	***–***	***–***	***ND*** 3	***–***	***–***	***–***	***–***	***–***	***–***	***–***	***–***	***–***	***ND*** 3	***–***	***–***	***–***	***–***	***–***
	Closed stable	*1*	*C. brunnicans*	2		1								1																		
		*2*	*C. obsoletus*	18	4	8	*25*	1		1	7			1					3	1												
		*3*	*C. scoticus*	2	2	1				1	1								1													
		*4*	*C. punctatus*	1																1												
		*8*	*C. chiopterus*	3	1			3											1													
			Obsoletus Complex	1													1															
	**Total Closed stable**			**27**	**7**	**10**		**4**		**2**	**8**			**2**			**1**		**5**	**2**		***–***	***–***	***–***	***–***	***–***	***ND*** 3	***–***	***–***	***–***	***–***	***–***
	**TOTAL**			**948**	**38**	**203**		**95**		**2**	**58**		**6**	**516**		**24**	**56**		**6**	**20**		**451**	**88**	**174**		**64**	**164**		**4**	**113**		**20**

1 R: species rank calculated with the total number of individuals whatever the trap and the season.

2 F: females; *(P):* parity rate as No. parous/No. females (given in percentage if F >5); M: males. For the sake of clarity, 0 were not quoted.

3 ND: not done. The drop trap was not used in the largely open stable, and only one UV-light/suction trap was used outdoors.

Diversity and abundance of species varied across seasons and traps. *Culicoides brunnicans* was present almost exclusively in spring (Table 1). *Culicoides obsoletus* was present during the three periods, mainly in summer (Table 1). The abundance of *C. scoticus* decreased progressively from the first to the last collection period (Table 1). *Culicoides brunnicans* was more abundant in host-baited traps than in the UV-light/suction trap, whereas the opposite situation was observed for *C. obsoletus*. Moreover, the UV-light trap collected some species rarely or not collected in host-baited traps, as *Culicoides punctatus* Latreille and *Culicoides festivipennis* Keiffer. On the contrary *C. dewulfi* and *C. chiopterus* were found in host-baited traps but not in the UV-light/suction trap (Table 1).

### Endo/exophagy

The best model predicting the observed abundance of *Culicoides* overall (Pearson’s product-moment correlation, *r = *0.98) was the complete model including location, season, interactions between both and trap as fixed effects and the session as a random effect, even though season lacked a clear effect on abundance (Table 2). Most *Culicoides* females were collected outdoors (mean predicted number of *Culicoides* was 8.5 outdoors *versus* between 0.4 and 1.0 indoors) and more were collected by the drop trap (4.2 *versus* 2.4).

**Table 2:** Mean No. observed (max) and predicted *Culicoides* for all species and the most abundant species depending on the trap, the location and the season.

Effect	Value	All species		*C. brunnicans*		*C. obsoletus*		*C. scoticus*		*C. dewulfi*	
		Observed	Pred^3^	Observed	Pred	Observed	Pred	Observed	Pred	Observed	Pred
**Trap**	Drop trap	16.4(249)	4.18^***^	13.4(237)	1.94^***^	2.1(19)	0.91^***^	0.2(4)	0.04^***^	0.1(1)	0.04^*^
	Suction trap	6.6(99)	**2.37**	2.1(63)	**0.45**	2.7(28)	**1.62**	0.8(25)	**0.22**	0.3(3)	**0.26**
**Location**	Outdoor	25.0(249)	8.46^***^	16.4(237)	3.55^***^	5.4(28)	3.09^***^	1.2(25)	0.34^***^	0.4(3)	**0.41**
	LOS^2^	1.2(6)	0.99^**^	0.1(1)	**0.03**	0.7(4)	**0.39**	0.2(2)	**0.04**	0.1(1)	**0.03**
	Indoor	0.8(6)	**0.38**	0.1(1)	**0.01**	0.5(5)	**0.32**	0.1(1)	**0.02**	0.0	**0.00**
**Season**	Spring	24.0(249)	5.62	19.6(237)	**3.55**	1.9(14)	**0.89**	1.3(25)	–	0.2(3)	–
	Summer	5.0(34)	2.03	0.2(3)	**0.04**	3.6(28)	**1.53°**	0.2(2)	–	0.1(0)	–
	Autumn	2.6(18)	2.17	0.0	**0.00**	2.0(14)	**1.38**	0.2(1)	–	0.2(2)	–
Sea*loc 1	–	p<0.001		–		p<0.001		–		–	

1 Interaction between season and location.

2 LOS: largely open stable; Pred: predicted values; NS: not significant.

3 Predicted values (Pred) are in bold and underlined if analyze of variance between models with and without that effect showed significant differences with for α = 0.05. P-values were given for effect modalities compared to a reference value, *i.e.* “suction trap” for trap, “indoor” for location and “autumn” for season (^***^p<0.001; ^**^p<0.01; ^*^p<0.05;^o^ p<0.1, no indication p>0.1).

The best model predicting *C. brunnicans* abundance (*r = *0.99) was the complete model without the interaction term (Table 2). This species was found almost exclusively during the first collection period and outdoors (between 100 and 300 fold more abundant outdoors than indoors) and mainly by the drop trap (1.9 *versus* 0.5 by the suction trap). Interaction between location and season (p<0.001) was needed to obtain the best prediction of *C. obsoletus* abundance (*r = *0.91). *Culicoides obsoletus* was collected more abundantly outdoors (between 8 and 10 fold more abundant outdoors than indoors) and by the suction trap (1.6 *versus* 0.9 by the drop trap). For *C. scoticus* and *C. dewulfi*, only trap and location as fixed effect were needed to obtain parsimonious models with good fit (*r = *0.98 and *r = *0.72). Both species were collected more abundantly by the suction trap (predicted abundance was 0.22 and 0.26 *versus* 0.04 and 0.04), and *C. scoticus* was collected more abundantly outdoors (between 9 and 22 fold more abundant outdoor than indoor).

### Circadian Host-seeking Activity

During collections, the time of sunrise ranged from 6h49 (GMT +1) the 26^th^ April to 8h06 the 8^th^ October, when the sunset ranged from 21h01 to 19h24 between the same dates.

**Figure 2:**
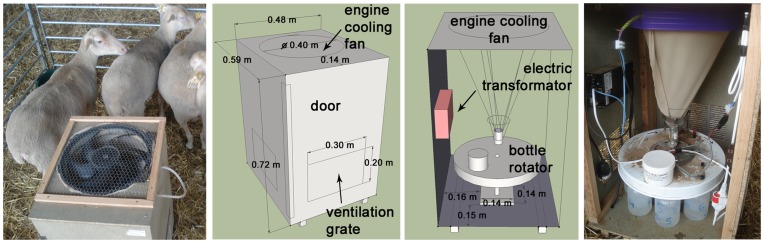
Circadian host-seeking activity of *C. brunnicans* and *C. obsoletus*: total number of females collected outdoor by host-baited traps at each session and day time. Small symbols are single collection and lines with large symbols the means by period. Vertical lines symbolize the time of sunrise and sunset.

In spring, *C. brunnicans* exhibited two peaks of diel activity. Host-seeking activity increased rapidly just after sunrise, and then slowly decreased during the next 5 hours. Host-seeking activity started again 5 hours before the sunset, reaching a peak just after sunset and then decreasing rapidly (Fig. 2).

Host-seeking activity of *C. obsoletus* was recorded mainly around sunset. In spring and autumn, host-seeking activity started slowly 2 hours before sunset, peaked just before sunset and then decreased rapidly (Fig. 2). In summer, we observed a similar pattern of host-seeking activity except that the peak occurred just after sunset. Minor host-seeking activity was recorded around sunrise, especially in autumn. Outside these 2 periods, host-seeking activity ceased. The surprising peak observed in the middle of the afternoon (15h20) was the result of a single collection with the drop trap.

The same pattern, *i.e.* host-seeking activity mainly around sunset and a small amount around sunrise, was observed for *C. dewulfi*, *C. chiopterus* and *C. scoticus*, even if the low number of females collected did not provide details of the daily host-seeking activity for these 3 species.

**Figure 3:**
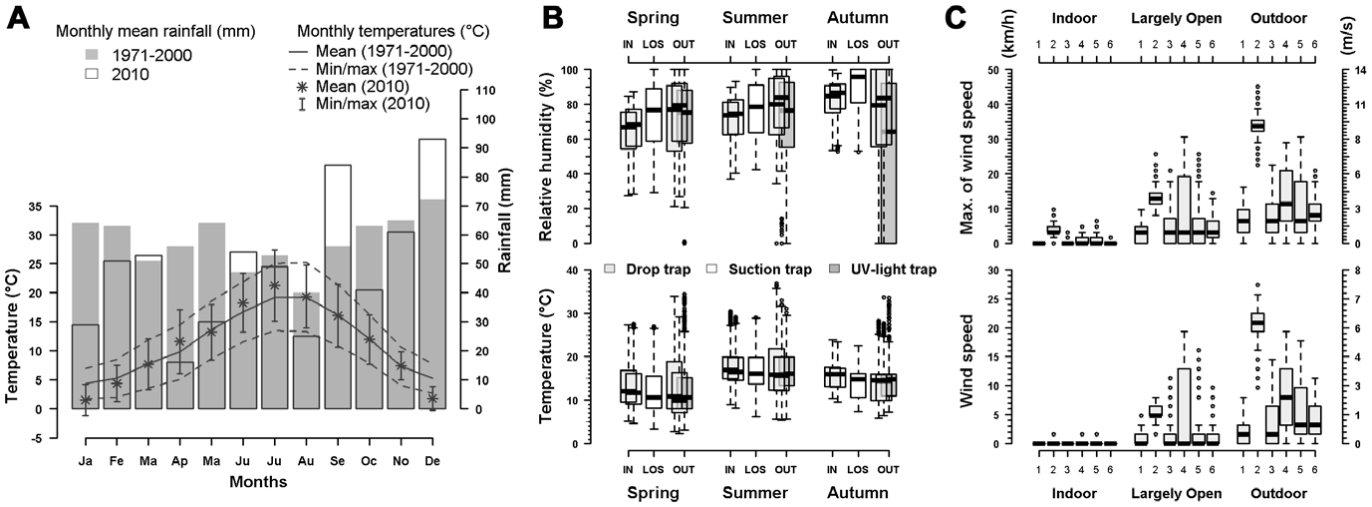
Number of *Culicoides* collected outdoor in host-baited traps compared to temperature and relative humidity recorded by data loggers. Lines are the distribution histogram of meteorological parameters recorded during the collections.

Almost all the collections were carried out without any rainfall. Comparing *Culicoides* abundance with data logger records, we noted that *Culicoides* were collected across a large range of temperature (Fig. 3): single individuals were collected at temperatures as lowest as 4.8°C and the first substantial catch (60 *Culicoides*) with low temperatures was recorded at temperatures between 8.4 and 10.4°C. Low relative humidity did not seem to inhibit host-seeking activity (Fig. 3) as 47 *Culicoides* were collected between 32 and 52% relative humidity. Moreover, we noted that the first significant catch (16 *Culicoides*) with strong wind was recorded with a wind speed of 5 m/s, but only 2/3 of collection sessions could be analyzed. Indeed, due to technical problems, local meteorological parameters were missing for some collection sessions.

**Figure 4:**
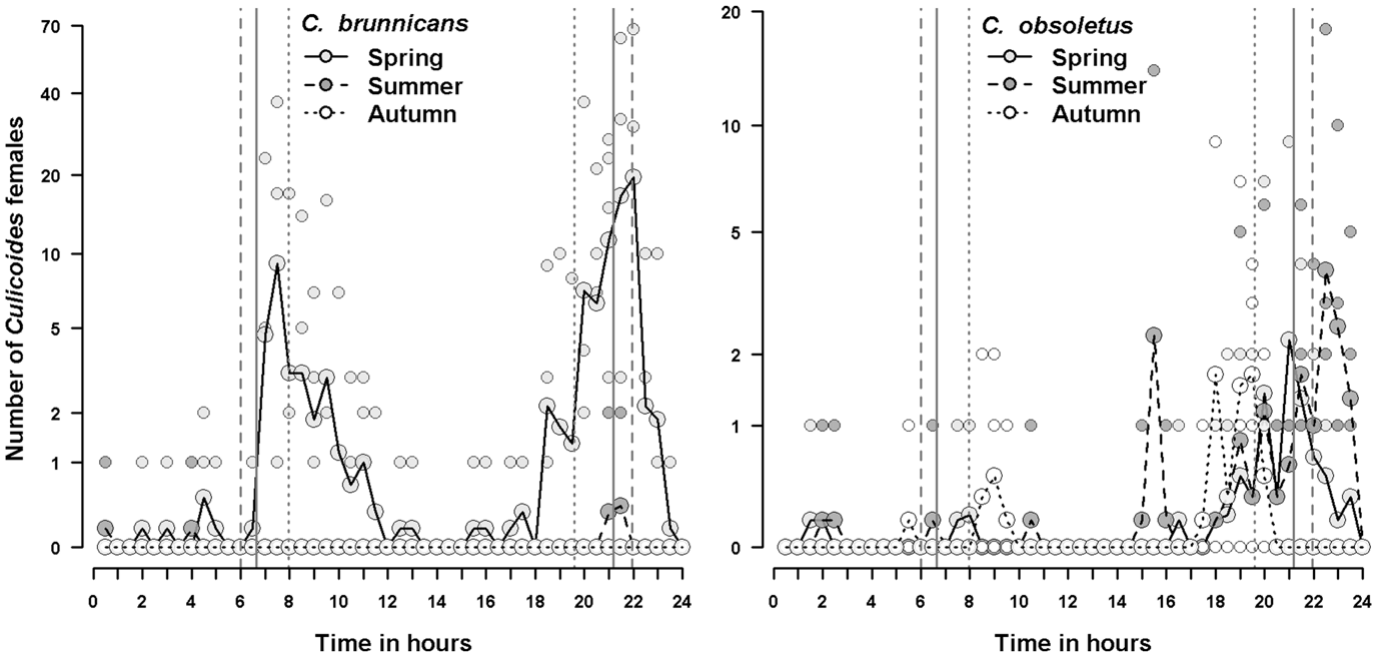
Correlation between the numbers of *C. brunnicans* and *C. obsoletus* collected outdoor in host-baited traps and in UV-light/suction trap.

*Culicoides brunnicans* biting rates in spring assessed by drop trap or suction trap were linearly and positively correlated with the abundance in the UV-light/suction trap (R^2^ = 0.55, p<0.001), but some collections were positive in host-baited traps but not in UV-light/suction trap, or conversely (Fig. 4). Correlation was much lower for *C. obsoletus* (R^2^ = 0.13, p = 0.032), although it seemed that UV-light/suction trap tended to over-estimate biting rates.

## Discussion

We described endo/exophagy and circadian host-seeking activity of Palaearctic *Culicoides* species using drop trap and suction trap collections. The mean number of *Culicoides* per collection was rather low. It illustrated the highly variable *Culicoides* abundance in space and time. Indeed, up to 3,000 *Culicoides* were collected at this site by single UV-light trap night-collections, carried out independently of these collections. The trap, as a fixed effect, was necessary to model abundances of all *Culicoides* species. The drop trap collected more *C. brunnicans* than the suction trap – Viennet *et al.* [16] showed that drop trap collected more *C. brunnicans* than other host-baited trap methods – but abundances assessed by both methods were highly correlated (Pearson’s *r* = 0.99). The suction trap collected more individuals of other species than the drop trap, and presence of gravid females suggested that this trap may have collected non host-seeking females. Moreover, differences between traps may reflect spatial variations of *Culicoides* populations. However, satisfactory concordance of abundance (*r = *0.67) for *C. obsoletus* in both traps suggested that the suction trap could be used as an efficient and easy alternative to drop trap for this species.

To date, investigations of *Culicoides* endo/exophagy had been carried out always with UV-light/suction traps [5], [6], [11]–[13], [21], [22], with the exception of one study [23] on ornithophilic *Culicoides* species. But UV-light/suction trap collections were greatly dependent on local conditions, such as presence and number of animals in the vicinity of trap [24], leading to complicated interpretations. For instance, *C. imicola* was collected in greater numbers by indoor animal-baited light traps than by outdoor unbaited light traps in Spain [21], in apparent contradiction with South-African descriptions, where this species exhibited exophagic behavior [5], [6]. In our study, host-baited collections in outdoor and indoor (with two degrees of opening for the host-baited suction trap) conditions highlighted that Palaearctic *Culicoides* are primary exophagous insects. Some species are strictly exophagous, such as *C. brunnicans*, some others show some degree of endophagy, such as *C. obsoletus* or *C. scoticus*. Baylis *et al.* [13] showed that differences between outdoor and indoor catches decreased from summer to autumn. To explain these differences, authors suggested factors difficult to untangle. Colder temperatures and stronger wind in autumn could suppress outdoor *Culicoides* host-seeking activity – overcast conditions have been shown to decrease outdoor UV-light/suction collections [6], [11]. On the other hand, autumnal conditions could also interfere in light trap efficiency – wind could modify the trap suction efficiency – or host-seeking activity could occur mainly before sunset late in the year, decreasing part of the *Culicoides* which otherwise would be collected [25], [26]. Even though interaction between season and location was necessary to model *C. obsoletus* abundance, our data did not allow to clearly confirm this observation. Finally, most of *Culicoides* females collected indoors in host-baited traps may have been host-seeking females. However, the low number of gravid *C. obsoletus* indoors suggest than some females may enter into sheds to oviposit, as illustrated by the possibility to find *Culicoides* larvae indoors [27], [28].

It is widely assumed that *Culicoides* are mostly crepuscular [29]. However, only few studies have described rigorously *Culicoides* circadian cycles with consecutive 24 h collections [18], [19], [30], and only one has considered host-seeking behavior of European *Culicoides* using horse-baited collections [20]. Two patterns of host-seeking activity were observed during our 24 consecutive hours of collection. *Culicoides brunnicans* showed a bimodal pattern of host-seeking activity with peaks at dawn and dusk, as found by Blackwell *et al.* [31] for flight activity of *C. impunctatus*. Other species, *C. obsoletus*, *C. scoticus*, *C. dewulfi* and *C. chiopterus*, showed a main activity peak around sunset, even if some individuals could be collected around sunrise. We cannot exclude that in context of higher *Culicoides* abundance, these species present a second, but lower peak of host-seeking activity around sunrise, as highlighted by Van der Rijt *et al.* [20] for *C. obsoletus* (84 individuals collected at sunset *versus* 10 at sunrise). Indeed, Sanders *et al.* [32] observed two peaks of flight activity for *C. obsoletus*, *C. scoticus*, *C. dewulfi* and *C. chiopterus* around both sunrise and sunset. Meteorological conditions are known to impact adult midge activity [29], [33]. It seems clear that windy conditions suppress the flight activity of *Culicoides* [6], [31], [33]. Flight activity is described experimentally as exceptional at temperatures below to 10°C and optimal at temperatures above to 20°C for *Culicoides oxystoma* Kieffer and *Culicoides pictipennis* (Staeger) [34]. Finally, Blackwell *et al.* [31] found a positive correlation between flight activity of *C. impunctatus* females and relative humidity and rainfall. During our observations, relative humidity was not found to influence host-seeking activity and minimal temperature required seemed in coherence with that described by Tsutsui *et al.* [34]. We found that the highest host-seeking activity occurred in low sunlight, corresponding to twilight periods. Changes in light intensity during the day could explain “abnormal” host-seeking activity. For instance, 15 *C. obsoletus* were collected in the middle of the afternoon (at 15h20) one day of June (Fig. 2). During this afternoon, sunlight decreased from 120 (14h00) to 77 (15h00) and to 20 J/cm^2^ (16h00), compared to means of 226 (14h00), 224 (15h00) and 225 (16h00) the other collection days of June. The first consequence is the possible lengthening of *Culicoides* attack hours when low-light overcast conditions prevail leading to biting throughout the day, as is known in open pastures for *C. impunctatus* or *C. obsoletus* and in forested environments for *C. obsoletus* [17], [35]. The other consequence is the possible nocturnal host-seeking activity of *Culicoides* due to moon illumination, as observed for North-American *Culicoides* species by Barnard and Jones [18] or Lillie *et al.* [19]. Therefore, it would be interesting to study influence of moon light and moon phases on circadian host-seeking activity of Palaearctic *Culicoides*. Sunset and sunrise times depend on locality and season, leading to a change in host-seeking activity times throughout the year. Moreover, *C. obsoletus* was active before sunset during spring and autumn, and after sunset during summer, illustrating the influence of other parameters than sunlight. This could be an adaptation to temperatures as suggested by Lillie *et al.* [19] who found the same pattern change throughout the year.

Finally, this work allowed the comparison of biting midge abundance in sheep-baited traps and in UV-light/suction traps, operated as a standardized method. Due to experimental design, these methods could not be rigorously compared. A positive and high correlation could be established for *C. brunnicans*, showing that UV-light collections may be used without correction to follow changes in biting rates. Correlation was worse for *C. obsoletus*, but the tendency of biting rate over-estimation by the UV-light/suction trap was already recorded by Carpenter *et al.* [14] and Viennet *et al.* [16].

To conclude, in farms with grazing animals, keeping valuable animals in closed stables would limit their risk to be bitten by *Culicoides* and then to be infected by *Culicoides*-borne pathogens, as biting midges seem to be primarily exophagic insects. However, practical recommendations are difficult to provide because of the variation in the degree of endophagy and time of host-seeking activity. The activity parameters depend on *Culicoides* species, host abundance, season, stabling conditions and weather. This illustrates the consequence of evolutionary processes selecting the ability of hematophagous insects to ensure host encounter in time and space.

## Materials and Methods

### Study Site and Culicoides Collections

**Figure 5:**
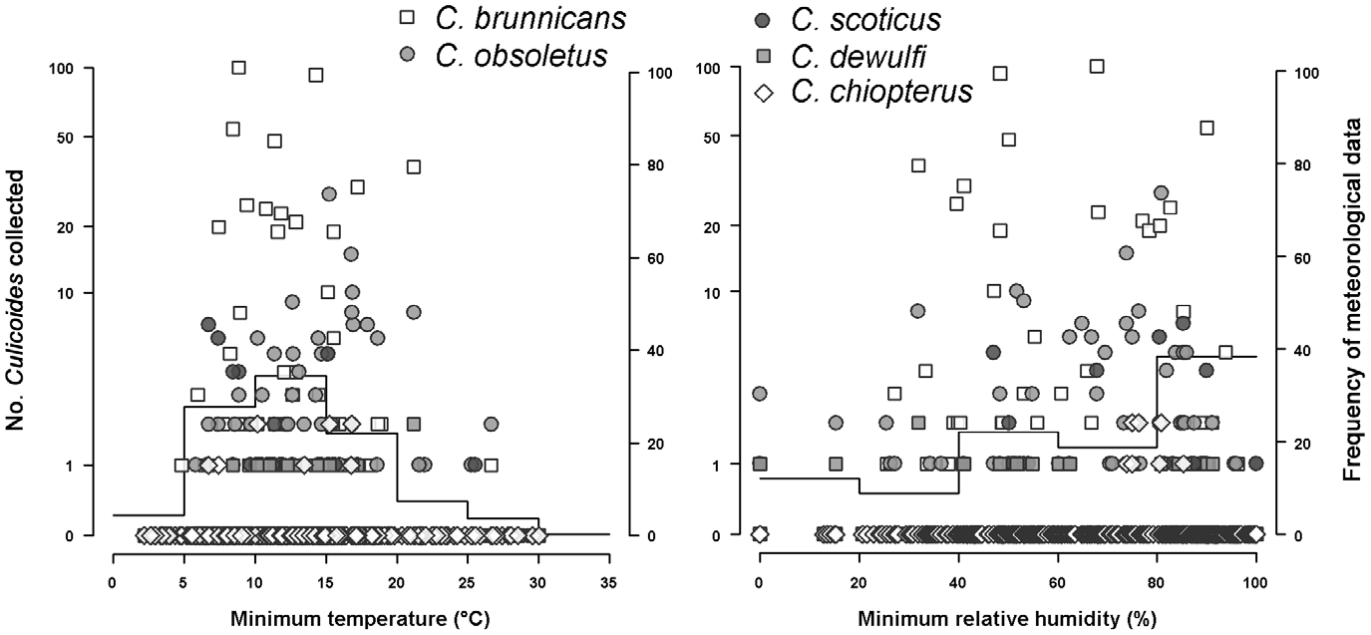
Sketch map of the study site at Nouzilly (western France) with the location of the sheep-baited traps (drop trap and suction trap) in the three sites (field, large-open-stable, closed stable) and of the UV-light/suction trap.

Indoor and outdoor collections were carried out during six collection periods of 24 hours in April/May, in late June and in September/October 2010 on an experimental farm (Institut National de la Recherche Agronomique, INRA, UE1277 PFIE) breeding sheep and located in Nouzilly (47°33′01′′N; 00°47′52′′E), western France. Three areas were investigated: a field (61×103 m), a closed stable (17×45 m) and a largely-open stable (30×10 m) (Fig. 5). The closed stable had closed windows (1×0.64 m; 30 windows west side and 12 east side) and two large doors (3.1×3.4 m) half open, thus about 3% of the walls were open surface. The largely open stable was constituted by 3 walls, 1 roof and 1 open side, and openings constituted about 38% of the wall surface. During collections, meteorological conditions (air temperature, relative humidity, wind speed and direction, solar radiation, rainfall and atmospheric pressure) were recorded every five minutes using a weather station Vantage Pro 2 (Davis Instruments France). Temperature and relative humidity were also recorded by a Tiny Tag TGP-4500 data logger at each trap location. Monthly meteorological data recorded by the national weather station (Météo-France) at Parcay-Meslay (47°26′36′′N; 00°43′36′′E; 13 km from the study site) in 2010 were used to illustrate meteorological conditions during the year of collections.

**Figure 6:**
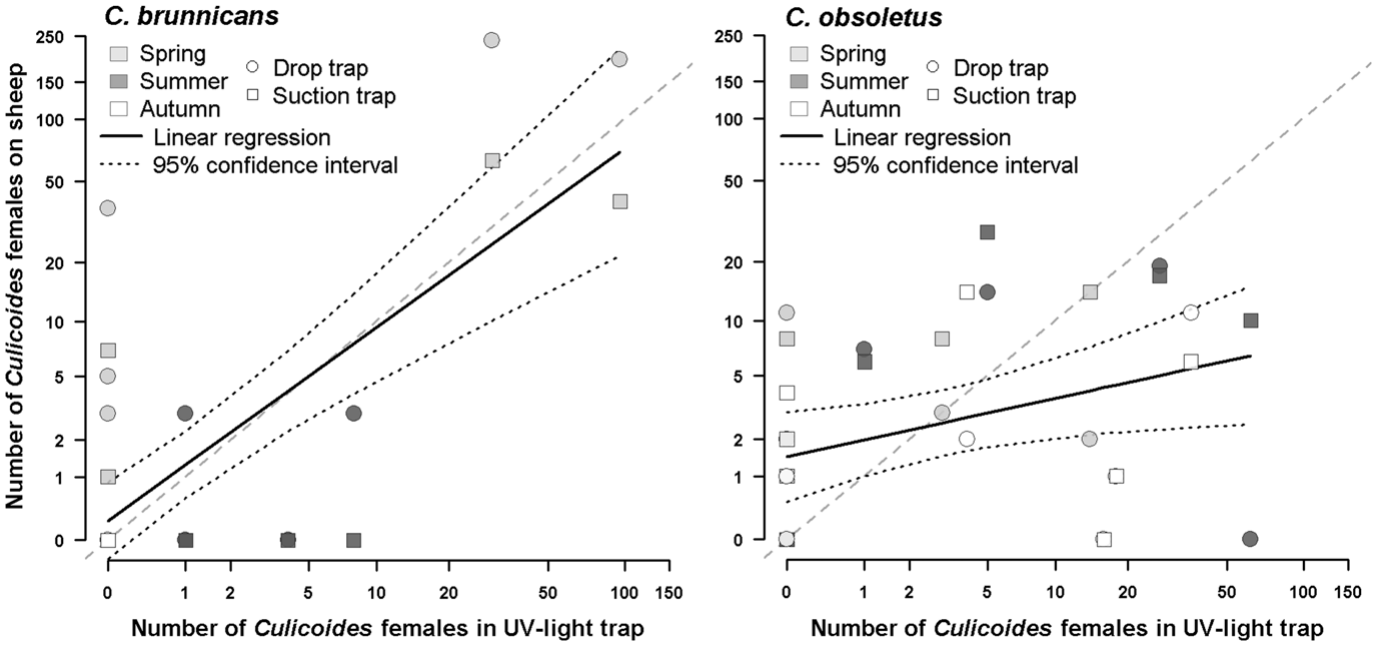
Inside and outside schemes and pictures of the suction trap.

We collected *Culicoides* using one drop trap and one suction trap in the field and in the closed stable, and one suction trap in the largely-open-stable (Fig. 5). All traps were separated by a minimum of 30 m to minimize interference and to assess the level of endophagy of *Culicoides*. The drop trap is a host-baited trap and consisted of a rectangular cage (2.5 m wide×3 m long and 2 m high) and made of white cotton netting (<0.25 mm^2^ mesh size). Its structure and use was described in detail by Viennet et al. [16]. The suction trap allows *Culicoides* collections without human intervention and consisted of a wooded box (48 cm wide×58 cm long and 73 cm high) equipped with a engine cooling fan at the top and an inner collection bottle rotator (model 1512, John W. Hock Company, Gainesville, FL) (Fig. 6). *Culicoides* were collected in 0.5 liter plastic collection bottles, which were filled 1/3 full with water and two drops of soap, and then were transferred in 70% ethanol.

Traps were operated during 24 consecutive hours (from 12 pm to 12 pm the next day), giving 48 collections at 30 min intervals, 6 times at each of the three 2-week periods. Each trap was baited with four south-Prealpes sheep females of 3 to 5-years old and 55 to 65 kg weight enclosed in a pen (2.5 m long×2 m wide×1.3 m high). There were no horses or cattle within 200 m from the sites during the collection sessions. The protocol was examined internally in the *Plate Forme d’Infectiologie Expérimentale* (UE1277 PFIE) by an internal group in charge of animal welfare, including veterinary surgeon and animal keepers. The protocol procedure did not cause any pain or stress, *i.e.* no injection, no biological sample, no surgery. Thus, according to the Directive 2010/63/EU on the protection of animals used for scientific purposes in Europe, it was not necessary to submit this protocol to an ethics committee and each step of the protocol was conducted with respect to the standard ethical rules: staff were qualified for animal experimentation and premises were licensed for experimentation.

In parallel, a UV-light/suction trap (manufactured by the Onderstepoort veterinary institute in South Africa) was operated during each collection session to provide an overview of *Culicoides* diversity. It was run with an 8 W UV light tube and on a 12-volt car battery, was placed at 1.5 m height from the ground on a tree and was not visible from the animal baits (Fig. 5). The insects collected with the UV-light/suction trap were stored in 70% ethanol.

### *Culicoides* Identification

All *Culicoides* were morphologically identified under a stereomicroscope (Stemi 2000 C ZEISS) to species level based on an identification key for the Palaearctic region [36] and sorted by sex. Females were classified as nulliparous, parous [37], freshly blood-fed and gravid. When morphological identification with a stereomicroscope was not possible, individuals were dissected and identified using microscopic slide preparations (ZEISS imager A.1 fluorescence microscope).

Individuals belonging to the Obsoletus Complex (*C. obsoletus* and *C. scoticus*) were molecularly identified following the assay developed by Nolan *et al.* [38]. DNA extraction was done high-throughput using Chelex100® resin (200 µL/*Culicoides*) before polymerase chain reaction (PCR) [39]. Primers and PCR amplifications conditions were as described by Nolan *et al*. [38].

### Statistical Analysis

Outdoor activity of *Culicoides* was compared between periods of the year to highlight influence of day length on circadian activity and was compared with climatic conditions to assess influence of meteorological parameters on host-seeking activity.

Abundance of *Culicoides* species was modeled using a Poisson mixed-effect model fitted with a method providing an adaptive Gauss-Hermite approximation to the log-likehood. We used the session (1 to 18) as a random effect, and the location (field, closed stable and largely-open-stable), the trap (drop trap, suction trap) and the season (spring, summer and autumn) as fixed effects. We considered also interactions between location and season, as endophagic behavior may change between the different seasons [12], [13]. Selection of effects and/or interactions in the abundance model was based on a likehood ratio test, and Pearson’s product-moment correlation was used as an overall test for goodness of fit.

Finally, we tested outdoor biting rates assessed by the host-baited traps with the abundance in UV-light collections using linear models.

All data analyses were performed using the R statistical package [40].
