# Correction: Host-Seeking Activity of Bluetongue Virus Vectors: Endo/Exophagy and Circadian Rhythm of *Culicoides* in Western Europe

**DOI:** 10.1371/annotation/a3b25de1-ec34-4777-9d16-0a60ef54a1a5

**Published:** 2013-04-04

**Authors:** Elvina Viennet, Claire Garros, Ignace Rakotoarivony, Xavier Allène, Laëtitia Gardès, Jonathan Lhoir, Ivanna Fuentes, Roger Venail, Didier Crochet, Renaud Lancelot, Mickael Riou, Catherine Moulia, Thierry Baldet, Thomas Balenghien

The figures in this article were placed incorrectly. The correct figures can be found below:

Figure 1: 

**Figure:**
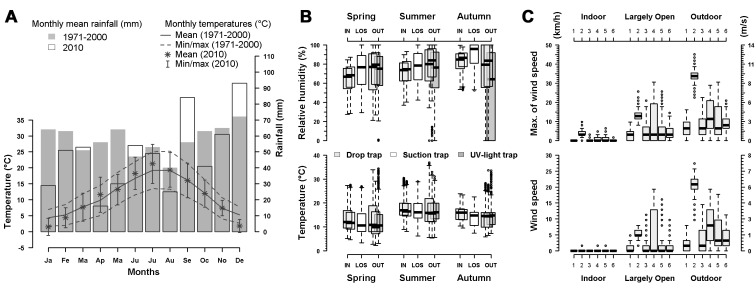


Figure 2: 

**Figure:**
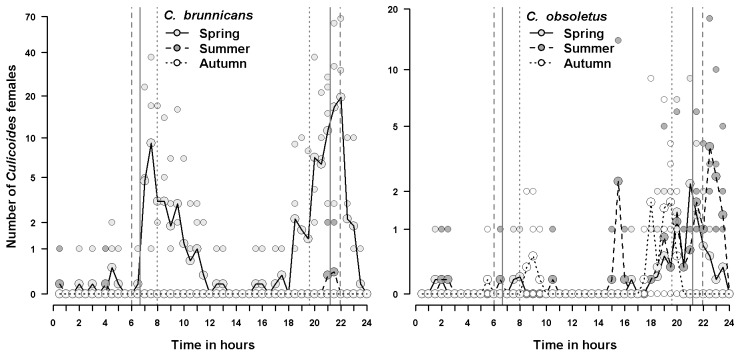


Figure 3: 

**Figure:**
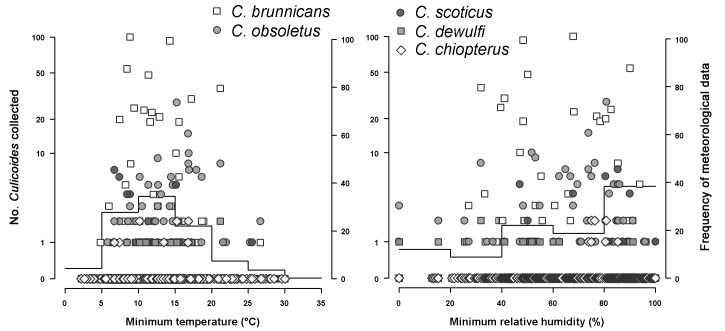


Figure 4: 

**Figure:**
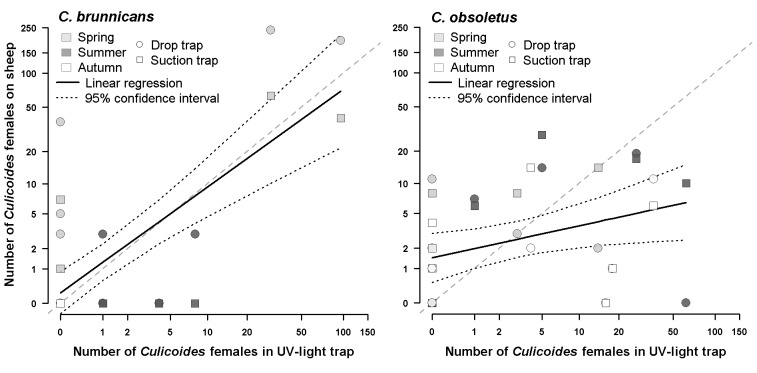


Figure 5: 

**Figure:**
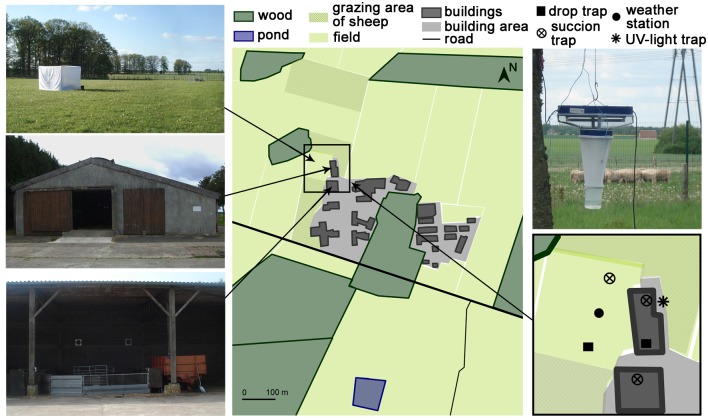


Figure 6: 

**Figure:**
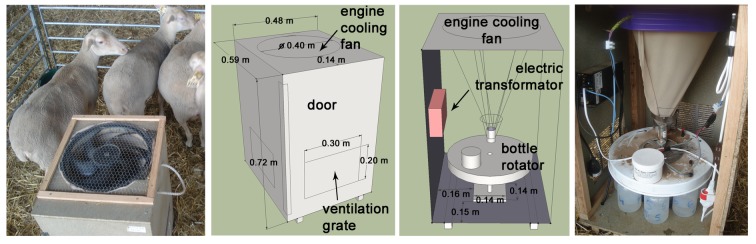


All legends are placed correctly.

